# Extracellular-Vesicle-Based Therapeutics in Neuro-Ophthalmic Disorders

**DOI:** 10.3390/ijms24109006

**Published:** 2023-05-19

**Authors:** Hamed Massoumi, Sohil Amin, Mohammad Soleimani, Bita Momenaei, Mohammad Javad Ashraf, Victor H. Guaiquil, Peiman Hematti, Mark I. Rosenblatt, Ali R. Djalilian, Elmira Jalilian

**Affiliations:** 1 Department of Ophthalmology and Visual Sciences, University of Illinois at Chicago, Chicago, IL 60612, USA; hmasso2@uic.edu (H.M.);; 2 The Richard and Loan Hill Department of Biomedical Engineering, University of Illinois at Chicago, Chicago, IL 60607, USA; 3 Wills Eye Hospital, Mid Atlantic Retina, Thomas Jefferson University, Philadelphia, PA 19107, USA; 4 Department of Medicine, Medical College of Wisconsin, Milwaukee, WI 53226, USA

**Keywords:** extracellular vesicles, exosomes, cornea, nerve innervation, neuro-ophthalmic, ocular surfaces

## Abstract

Extracellular vesicles (EVs) have been recognized as promising candidates for developing novel therapeutics for a wide range of pathologies, including ocular disorders, due to their ability to deliver a diverse array of bioactive molecules, including proteins, lipids, and nucleic acids, to recipient cells. Recent studies have shown that EVs derived from various cell types, including mesenchymal stromal cells (MSCs), retinal pigment epithelium cells, and endothelial cells, have therapeutic potential in ocular disorders, such as corneal injury and diabetic retinopathy. EVs exert their effects through various mechanisms, including promoting cell survival, reducing inflammation, and inducing tissue regeneration. Furthermore, EVs have shown promise in promoting nerve regeneration in ocular diseases. In particular, EVs derived from MSCs have been demonstrated to promote axonal regeneration and functional recovery in various animal models of optic nerve injury and glaucoma. EVs contain various neurotrophic factors and cytokines that can enhance neuronal survival and regeneration, promote angiogenesis, and modulate inflammation in the retina and optic nerve. Additionally, in experimental models, the application of EVs as a delivery platform for therapeutic molecules has revealed great promise in the treatment of ocular disorders. However, the clinical translation of EV-based therapies faces several challenges, and further preclinical and clinical studies are needed to fully explore the therapeutic potential of EVs in ocular disorders and to address the challenges for their successful clinical translation. In this review, we will provide an overview of different types of EVs and their cargo, as well as the techniques used for their isolation and characterization. We will then review the preclinical and clinical studies that have explored the role of EVs in the treatment of ocular disorders, highlighting their therapeutic potential and the challenges that need to be addressed for their clinical translation. Finally, we will discuss the future directions of EV-based therapeutics in ocular disorders. Overall, this review aims to provide a comprehensive overview of the current state of the art of EV-based therapeutics in ophthalmic disorders, with a focus on their potential for nerve regeneration in ocular diseases.

## 1. Introduction

**Figure 1:**
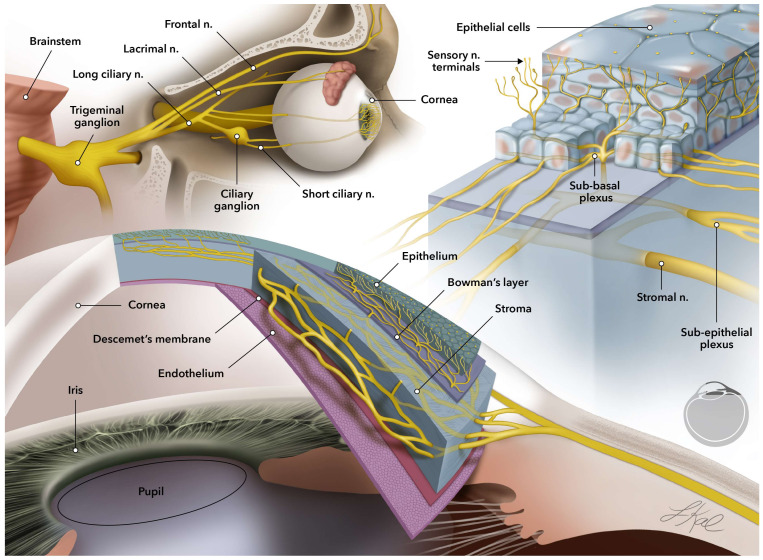
Schematic representation of corneal layers and sensory nerve innervation in the human eye. The cellular layers of epithelium, stroma, and endothelium are distinguished from each other by two acellular membranes of Bowman’s and Descemet’s membranes. Sensory neuron innervation is schematically presented in the cornea inlet. The sensory nerves are originated in the trigeminal ganglion and penetrate to the cornea through the stroma layer (stromal nerves). As the nerve branches climb up to the surface of the cornea, they start dividing into thinner neurites (subepithelial plexus) right before they traverse through Bowman’s membrane and divide into even thinner neurites through basal epithelium layer and finally, end in sensory terminals in the epithelium, which is the outermost layer of the cornea.

The cornea is the first layer of the eye, and it plays a crucial role in the vision process as the interface contact with the outside world [1]. Like the skin, which is the first line of defense against surrounding risk factors, the cornea, as the outermost layer of the eye, plays a pivotal role in ocular health [2]. The cornea covers one-sixth of the anterior part of the eyeball in humans and consists of multilayers of cellular (epithelium, stroma, and endothelium) and acellular (Bowman’s and Descemet’s membranes) components [2,3]. The cornea is the most densely innervated tissue in the body and the corneal nerves are responsible for temperature, touch, pain sensations, blink reflexes, tear production, and wound healing [4]. The nerve supply to the cornea is derived from the ophthalmic division of the trigeminal nerve. The corneal nerves form a dense subepithelial plexus that gives off numerous branches that penetrate the Bowman’s layer and enter the epithelium. The nerve terminals in the epithelium are specialized sensory receptors that detect mechanical and noxious stimuli (Figure 1) [4,5].

Damage to corneal nerves can result in lost or inadequate innervation and reduced corneal sensitivity, which can be evaluated through various methods such as touch using cotton wisp, air puff, or specialized instruments such as Cochet–Bonnet or Belmonte aesthesiometers. Other signs of corneal nerve damage may include changes to the corneal epithelium, such as decreased density, punctate erosions, thinning, impaired wound healing, and reduced tear secretion [6]. Additionally, nerve fibers can be directly visualized using confocal microscopy for a more detailed assessment of nerve structure and morphology [6]. Corneal nerves exhibit a limited capacity for endogenous regeneration at a slow pace after being damaged. Moreover, the sub-basal nerve will never fully recover to its original configuration, and current clinical treatment methods to respond to the loss of nerve innervation frequently suffer from low efficacy [7]. To develop more robust and effective treatments for neuro-ophthalmic diseases, researchers have tried many innovative therapies, such as exploiting stem cells and their secreting factors (i.e., EVs), to trigger nerve regeneration in ocular surfaces [8]. In this review article, we discuss innovative approaches and recent developments in isolating, characterizing, and using EVs in experimental and animal models as potentially novel therapeutics for neuro-ophthalmic disorders.

## 2. Current Treatments for Corneal Neurodegenerative Diseases

Current treatments for corneal neurodegenerative diseases are extremely limited and mainly focused on managing the symptoms and slowing the progression of the disease. One of the most used treatments is the application of artificial tears and lubricants to relieve dry eye symptoms. Additionally, medications such as cyclosporine and Lifitegrast have been approved for the treatment of dry eye by regulating the immune response and increasing tear production. In more severe cases, surgical procedures such as corneal nerve transplantation, limbal stem cell transplantation, and amniotic membrane transplantation, could be used to restore corneal sensitivity and improve ocular surface integrity [9,10]. These surgical-based interventions could lead to complications such as infection, tissue rejection, incomplete epithelization, and postoperative side effects [11]. Novel therapies such as nerve growth factors, neuroprotective agents, and gene therapy are also being investigated for their potential to promote corneal nerve regeneration and prevent further neurodegeneration [12]. 

Cell-based therapies and regenerative medicine have gained attention in the last two decades, because of their promising healing and therapeutic effects, potentially in a shorter period of time, with fewer complications and without the need for invasive and costly treatment [13,14,15,16]. Initially, the idea of exploiting stem cells’ abilities to regenerate indefinitely and differentiate into distinct types of cells seemed promising to treat nerve loss in ocular injuries. However, many of the original attempts resulted in mixed outcomes on account of the obvious drawbacks (i.e., poor engraftment, short-term storage stability, limited manufacturing capacity, immunogenicity, tumorigenicity, etc.) [17,18,19]. Indeed, currently, stem cells’ paracrine effects have become the subject of intensive studies, as mediators of nerve regeneration with the potential to replace the need for direct use of stem cells [20]. Osugi et al. observed that the media in which bone marrow mesenchymal stromal cells (BM-MSCs) were cultured, conditioned media, procured paracrine factors that could significantly impact the cell regeneration and healing process in rat calvarial bone defect [21]. Further investigations shed light on the presence of abundant soluble growth factors in conditioned media such as vascular endothelial growth factor (VEGF), platelet-derived growth factor (PDGF), fibroblast growth factor 2 (FGF-2), insulin-like growth factor 1 (IGF-1), brain-derived neurotrophic factor (BDNF), ciliary neurotrophic factor (CNTF), and nerve growth factor (NGF), all of which are hypothesized to be mediators in induced cell regeneration [22].

It is now well established that the bioactive constituents and secreted paracrine factors (secretome) are among the primary mechanisms through which stem cells exert therapeutic effects [16,23,24]. The term EV broadly encompasses the vesicle-based secretome components [25]. EVs are lipid membrane-bound vesicles of various sizes that are secreted from both eukaryotic and prokaryotic cells. EVs contain a wide range of cell-of-origin fingerprints such as DNA and RNA strands, lipid constituents, metabolites, proteins, etc. [26]. The impact of EVs extends from intracellular communication and maintaining homeostasis to promoting host-defense mechanisms and extracellular communicating tools by delivering cargo to target cells [27]. Many use the term “exosome” to refer to some or all EVs of a certain size, typically below 150 nm. Because until recently, the use of the term “exosome” was not standardized [28], and much high-value research was undertaken before this term became standardized, we will adopt the convention of using the term “small EVs” for exosomes and other small EVs approximately 150 nm or smaller for the remainder of this review. 

## 3. EV Characterization and Extraction Methods

**Figure 2:**
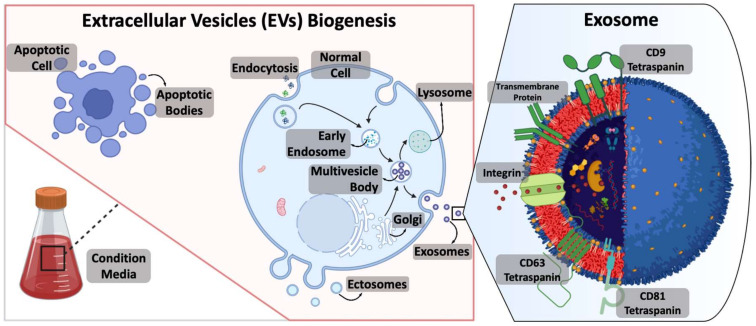
Biogenesis of EVs from apoptotic bodies or healthy cells incubated in a condition media. It is possible to predominantly classify EVs into three groups. Apoptotic bodies that are cell blebs produced at the time of apoptosis. Ectosomes, also known as microvesicles, are produced by the plasma membrane’s outward budding and dissociation. Exosomes are intracellular products of the endocytic membrane transport pathway interrupted by the fusion of multivesicular bodies with the plasma membrane. As depicted in the magnified inlet, exosomes are phospholipid bilayer containers encapsulating a variety of components (i.e., proteins, lipids, nucleic acids, metabolites, etc.) with multiple membrane receptors, proteins, and biomarkers.

EVs can be categorized into three groups based on their generation method and size: exosomes, microvesicles, and apoptotic bodies. Exosomes are the smallest (30–150 nm) and are produced within cells in multivesicular bodies (MVBs) that are the product of endocytosis and released upon the fusion of MVBs with the cell’s membrane. Some of the important biomarkers to distinguish exosomes from other EVs are the presence of tetraspanin proteins CD9, CD63, and CD81. Microvesicles are larger in range (100–1500 nm) and are produced by plasma membrane outward budding. The major biomarkers identified for these particles are CD40, integrins, and selectins. Apoptotic bodies are the largest (1–5 µm) and are produced by dying cells, and thrombospondin (TSP), histones, and C3b are the major biomarkers found in apoptotic bodies (Abs). This simplified categorization helps to understand the complex and heterogeneous nature of EVs and can be useful for targeted isolation and characterization strategies (Figure 2) [29].

There is a growing demand for reporting EV characteristics through robust and reliable laboratory techniques [30]. The International Society for Extracellular Vesicles (ISEV) proposed to define each prepared EV batch according to four criteria: (1) the source of the obtained EVs and its condition: The quantitative measures of the source (i.e., the secreting cells’ concentration, the mass of the source tissue, or the volume of body fluid in use) are noted as one of the EV characteristics. Furthermore, the incubation condition of the source (e.g., hypoxic, apoptotic, starving cells, etc.) can impact the secreted EVs and, therefore, must be reported and maintained for a uniform outcome. (2) EV concentration and abundance in the obtained medium; (3) EV-subtype-associated components depending on the level of specificity desired; and (4) the quality and quantity of the non-EV particles as well as EVs by measuring positive biomarkers associated with EVs and negative biomarkers associated with co-cultured compounds [27,31]. Particle count, size, and morphology, plus the total protein, lipid, and RNA contents, are typically reported as specifications of isolated EVs in published articles [32,33,34,35].

The characterization of EVs is crucial for downstream effect evaluations, including their therapeutic efficacy. However, common techniques such as dynamic light scattering (DLS) and flow cytometry may not accurately evaluate complex and multisize suspensions [36]. The total protein count can provide distinctive characterizations, but it cannot discriminate between co-isolated protein contaminants and usually leads to an overestimation of EV material [27]. The quantification of tetraspanins CD9, CD63, and CD81 can be used to determine exosome concentration in an extraction medium [37]. Currently, researchers mostly use single-particle tracking, protein concentration, Western blotting, and electron microscopy to characterize EVs [38]. Additionally, measuring the cargo and protein content of isolated EVs is likely to provide information about particle origin and may suggest its therapeutic capabilities. It is imperative to use multiple complementary characterization techniques to determine the uniqueness of isolated EVs [31].

To maximize the efficiency of EV-based therapeutics and increase production, it is crucial to have a precise and effective isolation technique that is easily expandable for up-scaled production while retaining the therapeutic potential of smaller-scale production runs. One of the most straightforward technologies for EV extraction is the use of ultracentrifugation, in which an ultrahigh-speed centrifugal force is used to separate EVs from both larger components (e.g., dead cells and debris) and smaller components (e.g., protein contaminants) [39,40,41]. The slight pitfall to this method of isolation is the dependency on highly sophisticated ultracentrifuges, which appears to be a minor issue [42]. This, and many other proposed methods for EV isolation and collection, are summarized in Table 1.

## 4. Therapeutic Applications of EVs

EVs obtained from MSCs have been shown to have therapeutic benefits, in various experimental models, such as enhancing wound healing, improving cardiac function after myocardial infarction, and reducing inflammation and fibrosis [39,50,51,52]. Using EVs instead of cells has advantages such as avoiding potential immune responses, ethical considerations, and the risks of transferring mutated or malignant cells [53]. EVs also allow for more accurate dosing since they lack proliferative properties, and their small size facilitates circulation and bypass of organ barriers that could trap cells [54].

**Figure 3:**
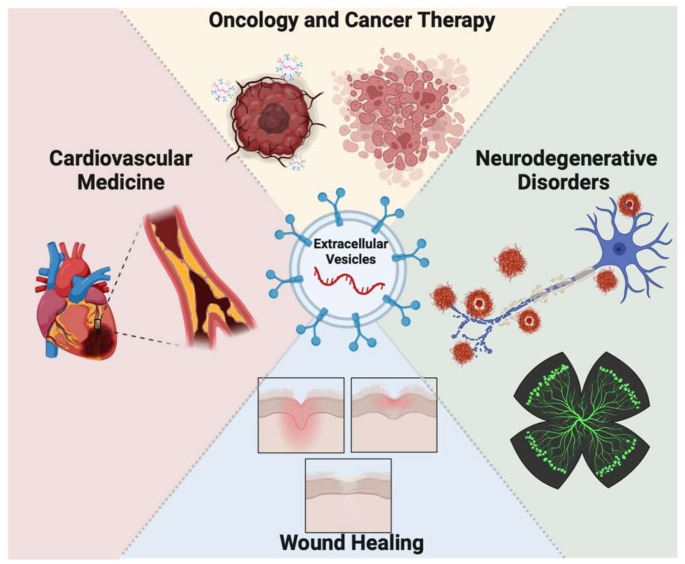
Therapeutic applications of EVs in clinical and preclinical studies published in the past decade are mostly focused on oncology and cancer therapy, cardiovascular medicine development, neurodegenerative diseases, and wound healing. The applications of EVs expand from diagnostic tools to novel therapeutics and can cover a wide range of organs and tissues.

The ISEV Annual Meeting presents new information each year on the biology and application of EVs, and oncology, cardiovascular medicine, neurodegenerative disorders, and wound healing are the most commonly studied topics (Figure 3) [55]. EVs derived from human sources have been investigated for their physiological and therapeutic effects in specific diseases within major categories, as EVs can be used as carriers for small RNA, anticancer drugs, and anti-inflammatory agents. Studies have shown that EVs can cross the blood–brain barrier and deliver their contents to target cells in the brain in animal studies [56,57].

EVs have the potential to be used for treating neurodegenerative diseases due to their ability to pass through the blood–brain barrier. Studies have shown that intranasal EV treatments have been effective in rodent models for Alzheimer’s, epilepsy, and other neurodegenerative diseases [58,59,60,61]. Additionally, microRNA (miRNA)-loaded exosomes could be used as a potential therapeutic option for multiple sclerosis (MS), with miR-219, miR-125a-3p, and miR-27a being potential targets for engineered EVs [62]. MSC-derived exosomes have been used to encapsulate curcumin and a magnetic resonance imaging contrast agent to treat Parkinson’s disease (PD), with miR-133b and miRNA-21 preventing neuronal death and reducing PD symptoms. Exosomes can also be used to regulate gene expression through exosome-derived miRNA, making them a promising therapeutic tool for inflammatory and neurodegenerative diseases in the brain and ocular surfaces [63,64].

In addition to the encapsulated therapeutic cargos, EVs have emerged as a promising therapeutic tool due to their unique surface chemistry that enables targeting capabilities [65]. According to a study by Kojima et al. [66], exosomes can be engineered to express specific surface proteins that allow them to target specific cells or tissues. The study demonstrated that exosomes expressing the surface protein Lamp2b were able to target and deliver therapeutic cargo to the brain, crossing the blood–brain barrier and reducing neuroinflammation in a mouse model of Alzheimer’s disease. The researchers also found that exosomes expressing the surface protein CD47 were able to evade phagocytosis by macrophages, increasing their circulation time in the bloodstream and enhancing their therapeutic efficacy [66]. Furthermore, other characteristics of EVs such as their stability in circulation and low immunogenicity profile make them a safe and effective therapeutic option [67].

### 4.1. Use of EVs for Ocular Neurodegenerative Diseases 

EVs are being investigated as a promising therapeutic approach for a range of ocular diseases, including corneal injuries, retinal degeneration, and glaucoma. Studies have shown that EVs derived from various cell types, including MSCs, retinal pigment epithelial (RPE) cells, and Schwann cells, can promote corneal wound healing, protect retinal cells from degeneration, and reduce intraocular pressure in glaucoma models [68,69]. EVs can also reduce inflammation, neuronal degeneration, and neovascularization. Patients suffering from eye disorders may receive more targeted medication if EVs are used as therapeutic carriers in their treatments [70]. 

In addition, EVs can be engineered to express or carry specific therapeutic molecules and can be targeted to specific ocular tissues or cells by modifying their surface properties. EV molecular profiles and cargoes are intriguing indicators for retinal and optic nerve injuries and illness, and they provide an adaptable and secure platform for delivering treatments to the tissues of the eye. Importantly, EVs can pass through corneal layers and enter host corneal keratocytes and endothelial cells [71]. The contained bioactive molecules can accelerate corneal proliferation, migration, and re-epithelialization [72] and enhance stromal cell proliferation and deposition of new collagen as well as other extracellular matrix (ECM) proteins [73]. Furthermore, reducing leukocyte infiltration, modulating matrix metalloproteinase activity, and alleviating apoptosis are other important mechanisms that make EV therapy an ideal alternative for ocular surface regeneration [69]. Therapeutic molecules dependent on MSC-derived EVs can regulate intercellular signaling pathways, and engineered EVs may be an emerging agent for different ocular diseases [72,74].

#### 4.1.1. Neurotrophic Keratitis

Neurotrophic keratitis is a spectrum of rare degenerative corneal diseases caused by the impairment of corneal and trigeminal sensory innervation, which leads to a decreased corneal sensation, epithelial breakdown, and ulceration. Herpes family viruses and topical anesthetic abuse are the most common causes [75]. General management involves promoting corneal healing and preventing ulcer development through the use of therapeutic patching, preservative-free artificial tears, topical autologous serum, and other lubricants [76]. Early in the disease process, topical corticosteroids may help in reducing corneal scarring. Recently, topical therapies provide recombinant human nerve growth factor and the restoration of ECM scaffolding properties through poly-carboxymethyl glucose sulfate (Cacicol), both of which improve corneal healing [77]. Refractory cases might need surgical approaches such as tarsorrhaphy, conjunctival flap, and amniotic membrane transplantation [78].

While we found no exact precedent in the literature, the delivery of EVs theoretically could slow the progression or improve the course of neurotrophic keratitis. The lack of nerve regeneration in the setting of neurotrophic keratitis may be due to neurotrophic growth factor (NGF) deprivation, which promotes neovascularization [79]. EVs could be used as a delivery vehicle for administering NGF. Yang et al. have demonstrated the therapeutic effects of NGF delivery via exosomes for cerebral ischemia [80]. They found that engineered exosomes containing NGF-coding mRNA reduced inflammation and promoted cell survival in vivo. The same principle could be applied for increased corneal nerve survival in the setting of neurotrophic keratitis and, potentially, any ophthalmic disease requiring neovascularization therapy. Furthermore, the potential effect of MSC-derived EVs on nerve regeneration has been shown by Jalilian et al. in vitro [7]. They showed that the EVs obtained from human BM-MSCs cultured in 2D and 3D microenvironments could enhance corneal nerve regeneration, and the effect was more significant when the EVs obtained from 3D-derived MSCs were used.

#### 4.1.2. Dry Eye Syndrome (DES)

DES is a result of an inability of the tear layer to properly lubricate the surface of the cornea. It has various causes, including diminished tear production and early evaporation of tears, and can occur in conjunction with more serious diseases such as corneal allograft rejection, graft vs. host disease (GVHD), and autoimmune diseases (most commonly Sjögren’s syndrome) [81]. It can occur secondary to factors disrupting nerves of the ocular surface such as iatrogenic corneal nerve damage following laser-assisted in situ keratomileusis (LASIK) or incisional nerve damage in creating wounds for cataract surgery [82,83].

Severe dry eye can lead to corneal ulceration and nerve damage. Primary management for dry eye syndrome is artificial tears, warm compresses, and limited topical steroid or cyclosporine use for anti-inflammatory effects [84]. In nonresponders, autologous serum tears may be used [85]. Novel methods utilizing anti-inflammatory mediators are also being explored, such as IL-1 antagonists, serine protease inhibitors, integrin antagonists, thrombospondin-1, and MSC therapy [86].

DES is closer to clinical viability for EV therapy than other eye diseases. MSC-derived EV treatment administrated as eye drop has progressed to clinical trial phases for the treatment of DES related to refractory chronic GVHD caused by corneal allograft transplantation [83]. A prospective clinical trial demonstrated substantial relief, quantified by increased tear production and reduced ocular surface index (OSDI) score [83]. This suggests that MSC-derived EVs have great potential in being efficacious agents for reducing inflammation and providing clinical benefits for dry-eye-related diseases. As EV treatment is an emerging therapy, these clinical trials are among the few examples of EV treatments that have progressed to this stage of viability to date. 

EVs derived from other stem cell phenotypes have been explored as well with regard to dry eye disease. A preclinical study investigated the therapeutic efficacy of EVs derived from human adipose tissue stem cells in the form of eye drops [87]. The eye drops were administered to mice with damaged ocular tissue from dry eye disease and were found to reduce inflammasome formation and IL-1B signaling, suppressing the inflammatory response and alleviating ocular surface damage. This study opens future applications of EV treatment derived from a variety of stem cell phenotypes for dry eye disease.

### 4.2. Retinal Diseases Causing Nerve Malfunctions

Neurodegenerative retinal diseases are among the most common and potentially blinding retinal disorders. Some well-known examples are diabetic retinopathies and genetic retinal diseases such as retinitis pigmentosa (RP) [88]. These diseases eventually lead to photoreceptor atrophy and the formation of a glial scar. Previous in vitro studies have shown the neuroprotective effects of MSCs in promoting retinal ganglion cell survival and decreased reactive gliosis [89]. However, the intravitreal injection of EVs for retinal injury is easier than MSCs and has a lower risk of vitreous opacification [90]. Stem-cell-derived EVs can penetrate the blood–retina barrier and are candidates to slow down photoreceptor degeneration and mitigate retinal injuries by changing the levels of inflammatory mediators [10]. Recently, the neuroprotective effect of human-MSC-derived EVs on reducing retinal photoreceptor degeneration has been demonstrated in vitro [91]. MSC-derived EVs also downregulate the expression of various inflammatory molecules, intercellular adhesion molecule 1 (ICAM1), VEGF-A, and monocyte chemoattractant protein 1 (MCP1) and therefore improve retinal injuries [92]. EV therapies are further investigated as promising candidates for retinal regeneration and neuroprotection by mediating intercellular communications [93].

#### 4.2.1. Diabetic Retinopathy (DR)

One of the most common retinal diseases in the US, diabetic retinopathy, is a microvascular complication of hyperglycemia, leading to microaneurysm formation and abnormal neovascularization that can progress to hemorrhage and blindness [94]. Diabetic retinopathy is one of the leading causes of blindness in adults worldwide. VEGF plays the most significant role in the pathogenesis of diabetic retinopathy by increasing the permeability of retinal capillaries. Currently, the mainstay of pharmacotherapy involves anti-VEGF drugs, including ranibizumab, pegaptanib, aflibercept, and off-label bevacizumab [95]. However, adverse effects and limitations, such as increased incidence of endophthalmitis, short half-life, and excessive cost provide hurdles [76]. Other therapeutic options include intravitreal steroids and laser photocoagulation. To replicate diabetic retinopathy, Maisto et al. stimulated retinal photoreceptors with high glucose concentrations. They discovered that high glucose levels lowered the amounts of antiangiogenic miRNA in photoreceptors and exosomes and raised VEGF levels [96].

The pathophysiology of diabetic retinopathy is overly complex, and Huang et al. demonstrated that plasma exosomes containing complement proteins may contribute to microvascular damage in diabetic retinopathy by activating the classical complement pathway. IgG-laden exosomes in plasma activate the complement system and their quantity is increased in diabetes. A lack of IgG in exosomes in diabetic mice results in reduced retinal vascular damage, suggesting that complement activation by IgG-laden plasma exosomes could play a role in the development of diabetic retinopathy [97]. The applicability of EVs loaded with specific cargo has also been studied with respect to diabetic retinopathy studies. Zhang et al. used MSC-derived exosomes loaded with exogenous miRNA-126 and administrated them via intravitreal injection into hyperglycemic rats with retinal inflammation [98]. It was found that the exosomes successfully carried their miRNA after both in vitro and in vivo applications and suppressed the pathway leading to retinal inflammation. Another study used a mouse model of diabetic retinopathy and studied adipose MSC-derived EVs through intravenous, subconjunctival, and intraocular injections [99]. Twelve weeks post-injection, subconjunctival and intraocular EV treatment enhanced retinal regeneration and the restoration of defined retinal compartments. In contrast, intravenous exosome treatment increased retinal thickness, with abnormal neovascularization. In addition, using rabbits as an animal model, the injection of EVs into the retinal tissue was shown to increase the expression of miRNA-222 and further supported the anti-inflammatory role of miRNA in the context of diabetic retinopathy [99,100]. 

Safwat et al. used intravenous (IV), subconjunctival (SC), and intraocular (IO) injections to deliver EVs from rabbit adipose-derived MSCs into mice eyes 4, 8, and 12 weeks after the diabetic mouse model induced by streptozotocin was established [99]. They found that exosome-encapsulated miR-222 can significantly reduce retinal edema and cell structural degradation in all layers of the retina. Additionally, they highlighted how exosomes control STAT5a, which modulates the responses of several cell ligands, including erythropoietin, thrombopoietin, and other growth hormones [101].

Li et al. recently reported that in high-glucose-treated cells, miR-486-3p is poorly expressed, while Toll-like receptor 4 (TLR4) and nuclear factor kappa B (NF-κB) were highly expressed [102]. Meanwhile, upregulating miR-486-3p or downregulating TLR4 inhibits diabetic retinopathy motivators such as oxidative stress, inflammation, and apoptosis. In this in vitro study, they showed that BM-MSC-derived EVs can play a protective role in diabetic retinopathy via TLR4/NF-κB axis repression by upregulating miR-486-3p expression from high-glucose-treated Muller cells.

#### 4.2.2. Retinitis Pigmentosa (RP)

RP is the most frequent hereditary retinal dystrophy, which is characterized by damage and the subsequent death of rod cells followed by cone cells. RP is a degenerative disease that still has no cure. However, as oxidative stress is one of the main pathologies in RP [103], the application of MSCs and their EVs might be a therapeutic option. The anti-inflammatory, antioxidant, and antiapoptotic properties of MSCs are helpful in targeting the underlying pathogenesis of the disease and slowing down retinal degeneration and RP progression. The trophic effect of the factors released by MSCs is shown to prolong the survival of photoreceptors, retinal Müller glia, and RPE cells [104].

The intravitreal injection of BM-MSC-derived EVs into a mouse model of RP had interesting results. The suppression of neuroinflammation evidenced by reduced expression of IL-6, TNFα, and IL-1β was noted. The degeneration of photoreceptors was retarded, with increased thickness of the retinal outer nuclear layer and improved electroretinogram and optokinetic tracking response [105]. However, the peribulbar injection of EVs obtained from allogeneic umbilical-cord-derived MSCs to four patients with severely damaged retinas due to late-stage RP did not show promising results [88]. Multifocal electroretinogram showed only minimal changes in photoreceptor activity in this group of patients up to six months post-injection [106].

#### 4.2.3. Optic Neuritis and Neuromyelitis Optica (NMO)

Optic neuritis can be either idiopathic or most commonly caused by multiple sclerosis, which results in the inflammatory demyelination of the optic nerve. Symptoms include episodic vision loss and painful eye movements. NMO includes the inflammation of both the optic nerve and the spinal cord. Oral steroids are the mainstay of medical management. Immune prophylaxis, including beta-interferons and glatiramer, has been used to lessen the progression of multiple sclerosis [107]. While steroids can sometimes improve the condition, there is no cure for optic neuritis, multiple sclerosis, and NMO. Few neuroprotective therapies exist, although a number of research groups have investigated LINGO-1 antagonists as a potential solution. LINGO-1 is a transmembrane cell surface glycoprotein, with roles in oligodendrocyte precursor cell and neuronal biology [108]. Clinical trials of a human aglycosylated monoclonal antibody blocking of LINGO-1 provided remyelination benefits in patients with less manifestation of ganglion layer thinning but found no improvement in patient disability outcomes so far [109]. Future studies may still find a clinical application for LINGO-1 antagonists.

EVs may exert potential treatment benefits by reducing the inflammatory response to prevent relapses in optic neuritis [110,111]. A practical example of engineered EVs in the setting of optic neuritis was demonstrated by Zhuang et al. Their group used macrophage-derived EVs complexed with curcumin, an anti-inflammatory dietary supplement, as an intranasal treatment in a mouse model of autoimmune encephalomyelitis [112]. They found that the treatment led to the rapid delivery of drugs encapsulated in EVs to the brain and increased uptake by microglial cells. This strategy serves as both a proof of concept for the delivery of engineered exosomes for demyelination injury and the therapeutic effectiveness of the exo-curcumin complex. These studies provide promising results for future applications to optic nerve demyelination injury. 

#### 4.2.4. Optic Neuropathy

The loss of oxygen or arterial supply can cause ischemic damage to the optic nerve, with a resulting clinical picture of sudden painless permanent vision loss. The two main types are anterior ischemic optic neuropathy (AION) and posterior optic neuropathy (PION), which are subdivided into arteritic and nonarteritic types [113]. Arteritic types require emergent high-dose corticosteroids to prevent vision loss [113]. However, these treatments provide no neuroprotection or reversal of existing ischemic damage to the optic nerve. Two clinical trials utilizing RPh201 and QPI-1007 have been examined in the setting of nonarteritic AION for their neuroprotective effects, with both showing modest improvement in visual acuity with treatment [114,115,116]. However, both have limitations in perceived clinical benefits and study designs.

EV delivery could offer neuroprotective benefits, with a similar mechanism to treatments of optic neuritis. Mead and Tomarev studied the effect of BM-MSC-derived EV administration in an optic nerve crush rat model, a model used in research to induce optic neuropathy [117,118]. They found that EV treatment promoted statistically significant survival of retinal ganglion cells and regeneration of axons. The proposed mechanism was the EV delivery of Argonaute-2, a mi-RNA effector [117]. Zhang et al. observed a similar result, with miR-126 enhancing neuroprotection and regeneration in the context of diabetic retinopathy [119]. Both of these studies demonstrate that EV administration has beneficial neuroprotective and axonal regeneration effects, with miRNA playing a key role in the signaling cascade.

#### 4.2.5. Glaucoma

Glaucoma is one of the most prevalent visual problems in the US. It is characterized by increased intraocular pressure that can lead to retinal ganglion cell atrophy, optic nerve damage, and visual field defects [120]. It is a frequent cause of the slow degeneration of retinal ganglion cells with several hypothesized mechanisms including inflammation, oxidative stress, neurotrophic factor deprivation, excitotoxicity, and antero-retrograde axon transport dysfunction. As one of the leading causes of permanent vision loss worldwide, many treatments have been tried and tested, though currently, the only efficacious modality is a reduction in intraocular pressure. The most commonly used therapeutics for this are topical beta-blockers, carbonic anhydrase inhibitors, prostaglandin analogs, and alpha-2 agonists [120]. Operative interventions include surgical or laser therapy to increase the outflow of aqueous humor and cryotherapy to reduce aqueous humor production [120]. These treatment options only function to slow progression and cannot reverse existing damage. Rho kinase inhibitors have shown promise as an emerging therapeutic, with a mechanism of enhancing aqueous drainage via the regulation of smooth muscle contraction through actin [121]. 

EVs in glaucoma patients could serve as both diagnostic markers and delivery vehicles for treatment. Dismuke et al. observed aqueous humor samples and noticed the presence of exosomes containing various miRNAs, such as miR-86-5p, miR-184, miR-204, and miR-182. Liu et al. subsequently found that miR-182 was highly expressed in the exosomes from trabecular meshwork cell origin and suggested that miR-182 may be involved in the disease progression of open-angle glaucoma [122]. Although these studies about the usage of EV-based miRNAs as a biomarker are in preliminary stages, they hold promise as a clinical diagnostic tool for the early detection of glaucoma, before intraocular pressure begins to rise.

In addition, researchers have loaded EVs with miRNA to increase the outflow of aqueous humor and protect the optic nerve. Mead et al. investigated BM-MSC-derived EVs as intravitreal therapy for rat models with glaucoma and found that the EV-treated group showed significant neuroprotection for retinal ganglion cells while preventing degenerative thinning and atrophy [63]. They additionally identified 43 miRNAs upregulated in BM-MSC-derived EVs compared with the fibroblast control group, which offered no neuroprotective effects. This suggests that great neuroprotective effects may be offered through miRNA-dependent mechanisms originating from exosomes. Maed et al. developed an animal model of chronic ocular hypertension and injected MSC-derived EVs intravitreally once a month for 9 months. They observed neuroprotective effects through a reduction in axonal degeneration and the preservation of retinal ganglions [123]. Mead et al. also studied the effect of MSC-derived exosomes in human retinal ganglion cultures in vitro, which showed great neuroprotective effects and was further enhanced via TNF-alpha signaling using etanercept [124]. These results confirmed the previous findings without any noted adverse effects. However, the duration of the neuroprotective effects can be questioned, as the second study reported the reduced efficacy of therapeutic benefits after 6 months. 

Another group recently studied the effects of mi-RNA-loaded exosomes in the context of the trabecular meshwork. Li et al. investigated the effect of BM-MSC-derived exosomes on trabecular meshwork cells, which allows for increased aqueous drainage, thus helping to alleviate elevated intraocular pressure [125]. Trabecular meshwork cells exposed to hydrogen-peroxide-mediated damage were pretreated with exosomes for 24 h. The treatment improved the survival rate compared with the control group. They additionally found 23 mi-RNAs differentially expressed in the trabecular meshwork cells (a network of specialized cells that is located in the anterior chamber of the eye), which were pretreated with exosomes compared with the control group of the same cells, which had no treatment [125]. This suggests that exosomes could play a role in the prevention and restoration of the trabecular meshwork in the setting of glaucoma.

## 5. Engineered EVs for Advanced Therapies

**Figure 4:**
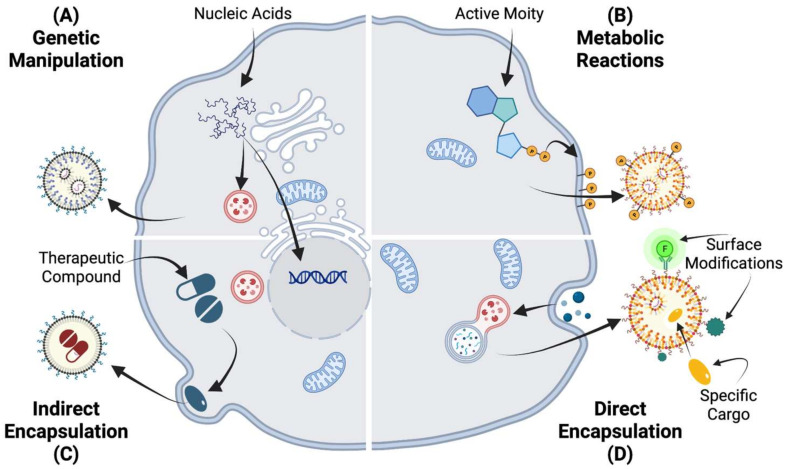
Engineering approaches in the modification of EVs can be categorized into four categories: (**A**) Coding and noncoding oligonucleotides can be introduced into cells through genetic engineering. Their genetic changes can impact the expression of different nucleic acid expressions in the EVs’ cargo. (**B**) Metabolic labeling is used for introducing non-native moieties into cells. In this technique, metabolite replicas are integrated into cell biosynthesis. (**C**) Exogenous materials can be introduced to the cells and indirectly load the secreting EVs. (**D**) Direct EV modification is to permeabilize the vesicle membrane to enable the active loading of EVs. The vesicle membrane itself can potentially be the site of chemical reactions.

Even though there are numerous experimental studies about the therapeutic effect of unmanipulated EVs used as therapeutics, the use of engineering EVs is being recognized as an innovative way to expand their therapeutic capacities [126]. There have been viral- or nonviral-based methods employed to engineer EVs that can be categorized based on their method of modification (Figure 4): (1) genetically altering parental cells to express targeting molecules; [57,127]; (2) changing the culture medium to trigger metabolic changes in parental cells to change the compositions of the secreting EVs [128]; (3) indirectly loading EVs with drugs (e.g., by culturing the cells in a drug-containing medium to uptake the moieties and load them in EVs through intracellular interactions) [129]; (4) directly loading EVs with drugs (e.g., using methods such as electroporation or sonoporation methods) [130].

There are potential advantages in using engineered EVs compared with other engineered therapeutic formulations such as whole-cell, single-molecule, or nanoparticle fabrications. High biostability and biocompatibility, ease of production and storage, and lower risk of malignant responses are a few advantages that can be exploited by using EVs for therapeutic means [131]. For instance, in an in vivo mouse model, Gong et al. demonstrated that angiogenesis was better promoted using EVs derived from genetically modified MSCs overexpressing GATA-4 (EV^GATA−4^) than those derived from MSCs transfected with an empty vector (EV^NULL^) [132]. It was found that EV^GATA−4^ contained higher levels of miRNAs from the let-7 family, compared with EV^null^. Upon EV^GATA−4^ transfer into HUVECs, a downregulation of thrombospondin-1 (THBS1) expression was recorded, which is a major endogenous angiogenesis inhibitor. Furthermore, Nkosi et al. demonstrated that the efficient secretion of latent membrane protein 1 (LMP1)-modified EVs promotes cell attachment, proliferation, migration, and tumor growth [133]. Acting as a viral protein, LMP1 alters EV content and changes the microenvironment of tumors by increasing the expression of ILV budding machinery. These observations provide insights into the LMP1 EV transportation mechanism and might propose novel engineered EV diagnostic and therapeutic techniques for the early recognition and prevention of cancers linked to the Epstein–Barr virus [133].

There are several methods for engineering the surface of EVs to achieve more functionalities, such as advanced targeting properties. These include genetic engineering, chemical modifications, and physical modifications such as sonication, extrusion, or the use of microfluidic devices for EV surface engineering [134].

As a genetic modification, in a study by Haney et al. [135], it was verified that the exosomes derived from MSCs could be engineered to express specific peptides that target the blood–brain barrier, enabling their uptake by brain endothelial cells and the subsequent delivery of therapeutic cargo to the brain. The study showed that MSC-derived exosomes expressing the RVG peptide were able to cross the blood–brain barrier and deliver siRNA to the brain, reducing the expression of a target gene in a mouse model of glioblastoma [135]. Furthermore, it has been proved that EVs derived from dendritic cells could be engineered to express specific surface proteins that target cancer cells [136]. The researchers found that EVs expressing the surface protein HER2 were able to target and deliver therapeutic cargo to HER2-positive breast cancer cells, reducing tumor growth in a mouse model of breast cancer [137]. Another study by Kooijmans et al. [138] revealed that EVs derived from dendritic cells (DCs) could be engineered to express specific surface proteins that target cancer cells. They pointed out that EVs expressing the surface protein EGFR were able to target and deliver therapeutic cargo to EGFR-positive cancer cells, reducing tumor growth in a mouse model of breast cancer. Moreover, EVs derived from MSCs could be engineered to express specific miRNAs that target cancer cells. The researchers showed that MSC-derived EVs expressing miR-146b were able to inhibit the growth and metastasis of lung cancer cells in a mouse model of lung cancer [139]. As a chemical modification, Zhu et al. investigated the effect of different formulations for culture media on the production of EVs by amniotic epithelial cells (AECs). The study aimed to identify the optimal culture conditions for maximizing EV production by AECs, which are promising sources of EVs for therapeutic applications [128]. The authors cultured AECs in different chemically defined media formulations and compared the yield and characteristics of EVs produced by the cells. The study found that the culture medium formulation had a significant impact on the yield and characteristics of EVs produced by AECs [128]. Finally, as a physical modification, Zhao et al. presented a microfluidic platform that integrates the processes of harvesting, antigenic modification, and release of surface-engineered exosomes into a single module. This streamlined approach enables the production of exosomes that are surface-engineered with MHC peptides without changing the intrinsic properties of the particles [134]. The researchers illustrated that by attaching melanoma tumor peptides such as gp-100, MART-1, and MAGE-A3 on the surface of exosomes, the antigen presentation and T=cell activation functions were greatly improved. They used exosomes that were engineered on the surface with murine antigen-presenting cells to activate gp100-specific CD8 T cells that were isolated from the spleen of two Pmel1 transgenic mice. As a result, the engineered exosomes induced significant proliferation of antigen-specific CD8 T cells, as compared to the native, unengineered exosomes [140].

EVs provide a natural vehicle for biomolecules because they are nanosized particles with low immunogenicity and low natural cytotoxicity, and are suitable for long-term storage. In addition, an understanding of mechanisms regulating cargo enrichment and EV release can be obtained from mechanical, biogenesis, and genomic studies. As evidence grows that EVs serve as critical components of basic and clinical studies, EVs are increasingly regarded as imperative mechanisms of intercellular communication [141]. There has been extensive research on the incorporation of proteins, RNAs, and lipids into EVs, but a gap remains concerning post-translational modifications and their importance in conferring specific properties to proteins. 

## 6. Future Directions of EV-Based Therapeutics in Ocular Disorders

The future directions of EV-based therapeutics in ocular disorders include several areas of research and development. First, there is a need to develop standardized protocols for the isolation and characterization of EVs, as this is crucial for ensuring the reproducibility and comparability of results across studies. Second, the identification of specific EV populations with the highest therapeutic potential for different ocular disorders is essential. Third, the optimization of delivery strategies for targeted and efficient therapeutic delivery is needed, as this will minimize the potential for off-target effects and enhance the efficacy of EV-based therapies. Fourth, the development of safe and efficient methods for scaling up the production of EVs for clinical use is also necessary. Fifth, the identification of optimal biomarkers for assessing the efficacy of EV-based therapies in clinical trials is crucial for monitoring treatment response and guiding the development of future therapies. Finally, the development of combination therapies involving EVs and other therapeutic modalities may enhance the therapeutic potential of EVs for ocular disorders. In summary, the future directions of EV-based therapeutics in ocular disorders involve addressing the challenges and opportunities associated with their development, with the ultimate goal of improving patient outcomes and quality of life. 

## 7. Discussion

New discoveries and understandings in cell biology and physiology have been revealed as a result of the identification and characterization of EVs [142]. Applications of EVs for diagnosis in clinical settings include disease recognition, monitoring disease progression, tracking treatment responses, and determining prognoses [143]. A number of EV-derived molecules have been proposed as biomarkers, but different EV collection techniques and analytical methods among researchers can create challenges in the validation processes. A prominent example is the isolation and detailed characterization of heterogeneous EV subpopulations. As a result of the development of these procedures, various useful outcomes will become available, including the identification of EV and sub-EV markers, along with the development of novel tools for the isolation of different types of EVs. Some questions still need to be addressed: How do EVs bind to target cells? How do they fuse and integrate with membranes? It is also crucial to understand the precise processes through which many of these ocular illnesses develop so that EV-based therapeutics could be developed to target particular pathways. Determining how the miRNA cargo in EVs changes in healthy and diseased individuals may reveal important distinctions and potential routes of action. Many ocular diagnoses are polygenic and impacted by several mechanisms [144]. Therefore, a better understanding of different diseases, especially neurodegenerative disorders, could also help in the future of the application of EVs in clinical practice.

Although the studies included in this review focused more on the significance of naturally occurring EVs, it must be emphasized that EVs provide the capacity of being altered and employed as organic nanocarriers. As exosome technology is emerging, there is an element of an experimental approach when extracting exosomes from various sources. As EV isolation currently lacks a gold standard, differences in isolation methods may have an impact on the results of research examining EV therapies [145]. In addition, there is also an unmet need for standardizing exosome quality and minimum standards of requirement before it can be clinically administered in patients [146]. The prospect of using EVs to treat neuro-ophthalmic disorders appears to be promising. However, whether these therapeutic results can be duplicated in human clinical trials has not yet been shown. Furthermore, the long-term efficacy and safety of these treatments need to be proven over the course of clinical trials before they can routinely be provided to patients with ocular disorders. The processes involved in EV extraction must be standardized, and global standards for EV quality assessments must be established [100]. 

## 8. Conclusions

This review paper was dedicated to providing a concise overview of EV usage in bench-top, preclinical, and some early-phase clinical models, as well as studies to treat a vast variety of disorders, especially ocular neurodegenerative diseases. Despite the need for improvement in many aspects of EV-based therapeutics, EVs have already demonstrated their potential applicability for combating diseases of the cornea, the retina, and the optic nerve, mainly in preclinical models. With the ever-increasing effort in designing innovative approaches, it is suggested that the utilization of exosomes and other subpopulations of EVs may have a significant impact on the future of therapeutics. We need more comprehensive in vivo investigations that might lead to human clinical studies despite the fact that interest in this sector of EV therapeutics is still growing. To the best of our knowledge, there are currently few clinical trials being conducted, but we anticipate that this will change in the future. EV treatments can be promising upgrades to the present patient treatments for lowering ocular disorders’ impact with prolonged efficacy.

## Figures and Tables

**Table 1:** Isolation techniques used to obtain EVs.

Isolation Method	Principal Mechanism	Equipment	Advantages	Disadvantages	Ref.
Differential centrifugation	Centrifugal force with ultrahigh-speed rotation	Centrifuge, ultracentrifuge, high-speed rotors, and rotary tubes	Straight forward, no additional chemical substance or reaction, high capacity, low processing cost, and high reproducibility	Expensive machinery and specialized equipment. Medium to high processing time (120–600 min)	[43,44]
Density gradient ultracentrifugation	Buoyant density in chemical solutions	Centrifuge, ultracentrifuge, high-speed rotors, and rotary tubes	Accurate particle separation, high capacity, minimum contamination chance	Expensive machinery and specialized equipment. High processing time (250 min–2 d)	[39,40,41]
Size exclusion chromatography	Separation of biomolecules based on their hydrodynamic radios	Porous beads	Prevention of EV aggregation and preserving their integrity. Low to medium processing time (1 mL/min)	Prospective contaminants of the target size and size overlap confusion. Low capacity	[45]
Commercial kit precipitation	Decreasing the solubility of EVs by polymer coating	Superhydrophobic polymers (e.g., PEG) and centrifugation	Simplicity, no need for further chemicals, and fast process (30–60 min)	Unspecified isolation, high polymer contamination, expensive, and low capacity	[46]
Immunoprecipitation	Antibody–tetraspanin bounding	Antibody-coated microbeads	Highly specified isolation, medium processing speed (240 min), high purity	Altered surface properties of EVs, difficult separation from beads, loss of EVs, expensive, and low capacity	[47]
Ultrafiltration	Filtration of assorted sizes	Porous membranes, and centrifugation	Highly specified sizes, simplicity, and fast processing (130 min)	Membrane plugging, unwanted contaminants, risk of low yield	[48]
Microfluidic	Entrapment of EVs by immunoaffinity or within porous structures	Microfluidic device	Highly specified isolation, simplicity, and fast processing for small sample size	Expensive machinery, small capacity, low yield	[49]

## Data Availability

Not applicable.
